# Supplementary material for: Postoperative joint pain is associated with long-term all-cause mortality after total joint arthroplasty
Source: PLoS One. 2025 Jul 3;20(7):e0327757. doi: 10.1371/journal.pone.0327757 (PMC12225861; doi:10.1371/journal.pone.0327757)
Supplement: S1 Table — Values are the mean ± standard deviation unless indicated otherwise. P-values were obtained by the Mann-Whitney U test or Chi-squared test wherever. *: P-value < 0.001. BMI: body mass index; TJA: total joint arthroplasty. (DOCX) [file pone.0327757.s001.docx]

**S1 Table. Patient characteristics of included and excluded TJA patients**

|  | **Included**  **(n=731)** | **Excluded**  **(n=261)** | *P-*  value |
| --- | --- | --- | --- |
| **Female (%)** | 55 | 49 | 0.14 |
| **Age at surgery (yrs)** | 64.81 ± 8.77 | 67.35 ± 12.70 | <0.001* |
| **BMI (kg/m^2^)** | 33.57 ± 6.95 | 31.81 ± 8.07 | <0.001* |

Values are the mean ± standard deviation unless indicated otherwise. *P*-values were obtained by the Mann-Whitney *U* test or *Chi*-squared test wherever. *: *P*-value < 0.001. BMI: body mass index; TJA: total joint arthroplasty.
