# Supplementary material for: Postoperative joint pain is associated with long-term all-cause mortality after total joint arthroplasty
Source: PLoS One. 2025 Jul 3;20(7):e0327757. doi: 10.1371/journal.pone.0327757 (PMC12225861; doi:10.1371/journal.pone.0327757)
Supplement: S2 Table — Values are p values obtained with Chi-squared test or Fisher’s exact test comparing each pain group with controls. Values smaller than 0.05 are marked in bold. (DOCX) [file pone.0327757.s002.docx]

**S2 Table.** **Comparison of prevalence of comorbidities and percentage of patients with multiple comorbidities between pain groups and controls ^#^**

| **Knee + Hip** | | | | | | | |
| --- | --- | --- | --- | --- | --- | --- | --- |
| Comorbidity | Controls | Sustained pain | P value | Pain while active | P value | Pain at rest | P value |
| Hypertension | 51% | 43% | 0.240 | 44% | 0.175 | 43% | 0.206 |
| Cardiovascular disease | 51% | 49% | 0.746 | 54% | 0.714 | 49% | 0.740 |
| Asthma | 10% | 4% | 0.182 | 5% | 0.150 | 5% | 0.156 |
| Hay fever | 4% | 1% | 0.495 | 2% | 0.400 | 2% | 0.756 |
| Eczema | 6% | 6% | 1.000 | 6% | 1.000 | 8% | 0.553 |
| Sinusitis | 10% | 10% | 1.000 | 8% | 0.625 | 12% | 0.714 |
| Heartburn | 34% | 29% | 0.533 | 29% | 0.355 | 33% | 0.884 |
| Irritable bowel syndrome | 8% | 10% | 0.665 | 9% | 0.973 | 10% | 0.563 |
| Crohn’s disease | 1% | 3% | 0.191 | 4% | **0.029** | 2% | 0.263 |
| Diabetes | 17% | 21% | 0.520 | 21% | 0.387 | 19% | 0.767 |
| Clinical depression | 4% | 15% | **0.002** | 11% | **0.010** | 13% | **0.004** |
| Anxiety | 8% | 15% | 0.117 | 11% | 0.393 | 15% | 0.060 |
| Epilepsy | 1% | 0% | 1.000 | 2% | 0.307 | 0% | 1.000 |
| Stroke | 2% | 1% | 1.000 | 2% | 1.000 | 1% | 1.000 |
| Motion sickness | 9% | 7% | 0.927 | 7% | 0.683 | 7% | 0.790 |
| Migraine | 10% | 19% | **0.031** | 18% | **0.016** | 16% | 0.098 |
| Cancer | 7% | 7% | 1.000 | 9% | 0.475 | 6% | 0.872 |
| Gout | 11% | 15% | 0.521 | 14% | 0.557 | 13% | 0.809 |
| Pager's disease | 0% | 0% | 1.000 | 0% | 1.000 | 0% | 1.000 |
| Bunions | 5% | 6% | 0.780 | 4% | 0.786 | 5% | 1.000 |
| Frozen shoulder | 7% | 6% | 1.000 | 8% | 0.997 | 5% | 0.493 |
| Osteoporosis | 18% | 24% | 0.307 | 23% | 0.214 | 22% | 0.396 |
| Carpal tunnel | 11% | 12% | 1.000 | 12% | 0.897 | 9% | 0.772 |
| Tennis elbow | 9% | 15% | 0.190 | 14% | 0.162 | 14% | 0.202 |
| Golfer's elbow | 1% | 4% | 0.076 | 3% | 0.221 | 5% | **0.041** |
| Acne | 2% | 0% | 0.606 | 1% | 1.000 | 0% | 0.609 |
| Viral warts | 2% | 0% | 0.606 | 0% | 0.362 | 0% | 0.609 |
| Cold sores | 9% | 6% | 0.638 | 9% | 0.899 | 6% | 0.526 |
| Hearing loss | 17% | 19% | 0.764 | 21% | 0.304 | 20% | 0.607 |
| Tinnitus | 7% | 10% | 0.539 | 9% | 0.804 | 10% | 0.435 |
| Glaucoma | 2% | 1% | 1.000 | 2% | 1.000 | 1% | 1.000 |
| Cataract | 17% | 9% | 0.137 | 11% | 0.179 | 12% | 0.310 |
| Myopia | 11% | 6% | 0.213 | 9% | 0.463 | 5% | 0.058 |
| Age-related macular degeneration | 3% | 4% | 0.416 | 3% | 1.000 | 5% | 0.284 |
| Incontinence | 8% | 12% | 0.401 | 10% | 0.525 | 9% | 0.827 |
| Polycystic ovary syndrome | 2% | 6% | **0.040** | 3% | 0.249 | 5% | 0.077 |
| > 2 comorbidities | 74% | 69% | 0.512 | 72% | 0.761 | 71% | 0.686 |
| > 3 comorbidities | 63% | 59% | 0.543 | 62% | 0.780 | 60% | 0.682 |
| > 4 comorbidities | 49% | 51% | 0.746 | 55% | 0.272 | 52% | 0.595 |
| > 5 comorbidities | 37% | 37% | 1.000 | 40% | 0.641 | 36% | 0.914 |
| > 6 comorbidities | 24% | 31% | 0.276 | 32% | 0.074 | 28% | 0.517 |
| > 7 comorbidities | 17% | 26% | 0.075 | 22% | 0.214 | 23% | 0.196 |
| > 8 comorbidities | 11% | 21% | **0.028** | 15% | 0.300 | 16% | 0.181 |
| > 9 comorbidities | 7% | 9% | 0.664 | 6% | 0.978 | 7% | 1.000 |
| > 10 comorbidities | 4% | 7% | 0.266 | 5% | 0.641 | 6% | 0.521 |
| **Knee** | | | | | | | |
| Comorbidity | Controls | Sustained pain | P value | Pain while active | P value | Pain at rest | P value |
| Hypertension | 54% | 49% | 0.637 | 48% | 0.412 | 49% | 0.599 |
| Cardiovascular disease | 53% | 44% | 0.348 | 52% | 0.932 | 45% | 0.366 |
| Asthma | 11% | 7% | 0.602 | 7% | 0.359 | 5% | 0.240 |
| Hay fever | 4% | 0% | 0.377 | 1% | 0.321 | 0% | 0.230 |
| Eczema | 6% | 7% | 0.733 | 7% | 0.834 | 9% | 0.369 |
| Sinusitis | 11% | 7% | 0.599 | 6% | 0.223 | 7% | 0.632 |
| Heartburn | 35% | 28% | 0.416 | 29% | 0.316 | 33% | 0.806 |
| Irritable bowel syndrome | 8% | 7% | 1.000 | 7% | 0.944 | 5% | 0.598 |
| Crohn’s disease | 1% | 5% | 0.098 | 6% | **0.009** | 4% | 0.144 |
| Diabetes | 19% | 28% | 0.266 | 25% | 0.293 | 25% | 0.387 |
| Clinical depression | 5% | 14% | **0.046** | 11% | 0.082 | 13% | 0.054 |
| Anxiety | 8% | 9% | 0.770 | 8% | 1.000 | 11% | 0.687 |
| Epilepsy | 1% | 0% | 1.000 | 1% | 0.481 | 0% | 1.000 |
| Stroke | 2% | 2% | 1.000 | 1% | 1.000 | 2% | 1.000 |
| Motion sickness | 9% | 5% | 0.403 | 4% | 0.117 | 5% | 0.448 |
| Migraine | 11% | 21% | 0.110 | 20% | **0.037** | 18% | 0.207 |
| Cancer | 8% | 7% | 1.000 | 10% | 0.773 | 5% | 0.783 |
| Gout | 12% | 19% | 0.298 | 14% | 0.622 | 16% | 0.455 |
| Pager's disease | 1% | 0% | 1.000 | 0% | 1.000 | 0% | 1.000 |
| Bunions | 7% | 7% | 1.000 | 5% | 0.624 | 5% | 1.000 |
| Frozen shoulder | 7% | 7% | 1.000 | 8% | 0.913 | 5% | 0.781 |
| Osteoporosis | 16% | 26% | 0.201 | 25% | 0.085 | 24% | 0.264 |
| Carpal tunnel | 13% | 12% | 1.000 | 13% | 1.000 | 9% | 0.514 |
| Tennis elbow | 9% | 16% | 0.224 | 14% | 0.211 | 15% | 0.310 |
| Golfer's elbow | 1% | 2% | 0.379 | 1% | 0.583 | 2% | 0.452 |
| Acne | 1% | 0% | 1.000 | 1% | 1.000 | 0% | 1.000 |
| Viral warts | 2% | 0% | 1.000 | 0% | 0.354 | 0% | 0.600 |
| Cold sores | 9% | 7% | 1.000 | 10% | 0.911 | 5% | 0.598 |
| Hearing loss | 16% | 23% | 0.366 | 24% | 0.140 | 22% | 0.428 |
| Tinnitus | 8% | 7% | 1.000 | 7% | 1.000 | 5% | 0.782 |
| Glaucoma | 2% | 2% | 0.568 | 2% | 0.658 | 2% | 1.000 |
| Cataract | 16% | 12% | 0.655 | 14% | 0.836 | 16% | 1.000 |
| Myopia | 12% | 5% | 0.202 | 8% | 0.423 | 4% | 0.064 |
| Age-related macular degeneration | 3% | 2% | 1.000 | 1% | 0.700 | 2% | 1.000 |
| Incontinence | 8% | 14% | 0.335 | 11% | 0.586 | 11% | 0.687 |
| Polycystic ovary syndrome | 2% | 5% | 0.222 | 2% | 0.658 | 4% | 0.307 |
| > 2 comorbidities | 74% | 72% | 0.878 | 75% | 1.000 | 73% | 0.912 |
| > 3 comorbidities | 66% | 63% | 0.777 | 65% | 0.936 | 62% | 0.622 |
| > 4 comorbidities | 51% | 53% | 0.858 | 58% | 0.299 | 53% | 0.897 |
| > 5 comorbidities | 40% | 40% | 1.000 | 43% | 0.684 | 36% | 0.698 |
| > 6 comorbidities | 28% | 33% | 0.642 | 35% | 0.255 | 29% | 0.978 |
| > 7 comorbidities | 19% | 26% | 0.448 | 22% | 0.746 | 22% | 0.808 |
| > 8 comorbidities | 12% | 19% | 0.361 | 13% | 0.963 | 15% | 0.808 |
| > 9 comorbidities | 7% | 7% | 1.000 | 5% | 0.624 | 5% | 1.000 |
| > 10 comorbidities | 4% | 5% | 1.000 | 4% | 1.000 | 4% | 1.000 |
| **Hip** | | | | | | | |
| Comorbidity | Controls | Sustained pain | P value | Pain while active | P value | Pain at rest | P value |
| Hypertension | 45% | 32% | 0.292 | 32% | 0.222 | 32% | 0.242 |
| Cardiovascular disease | 48% | 56% | 0.612 | 59% | 0.349 | 55% | 0.633 |
| Asthma | 7% | 0% | 0.368 | 0% | 0.222 | 3% | 0.697 |
| Hay fever | 4% | 4% | 1.000 | 3% | 1.000 | 6% | 0.626 |
| Eczema | 6% | 4% | 1.000 | 3% | 1.000 | 6% | 0.697 |
| Sinusitis | 7% | 16% | 0.238 | 12% | 0.488 | 19% | 0.073 |
| Heartburn | 31% | 32% | 1.000 | 29% | 0.992 | 32% | 1.000 |
| Irritable bowel syndrome | 7% | 16% | 0.238 | 12% | 0.488 | 19% | 0.073 |
| Crohn’s disease | 1% | 0% | 1.000 | 0% | 1.000 | 0% | 1.000 |
| Diabetes | 11% | 8% | 1.000 | 9% | 1.000 | 6% | 0.541 |
| Clinical depression | 3% | 16% | **0.024** | 12% | 0.059 | 13% | **0.045** |
| Anxiety | 8% | 24% | **0.031** | 18% | 0.149 | 23% | **0.030** |
| Epilepsy | 1% | 0% | 1.000 | 3% | 0.413 | 0% | 1.000 |
| Stroke | 2% | 0% | 1.000 | 3% | 0.590 | 0% | 1.000 |
| Motion sickness | 7% | 12% | 0.407 | 15% | 0.162 | 10% | 0.476 |
| Migraine | 7% | 16% | 0.120 | 12% | 0.300 | 13% | 0.269 |
| Cancer | 5% | 8% | 0.631 | 9% | 0.416 | 6% | 0.672 |
| Gout | 10% | 8% | 1.000 | 12% | 0.762 | 6% | 0.745 |
| Bunions | 3% | 4% | 0.554 | 3% | 1.000 | 3% | 1.000 |
| Frozen shoulder | 7% | 4% | 1.000 | 6% | 1.000 | 3% | 0.697 |
| Osteoporosis | 20% | 20% | 1.000 | 18% | 0.948 | 19% | 1.000 |
| Carpal tunnel | 7% | 12% | 0.427 | 9% | 0.728 | 10% | 0.714 |
| Tennis elbow | 9% | 12% | 0.475 | 12% | 0.520 | 13% | 0.497 |
| Golfer's elbow | 2% | 8% | 0.118 | 6% | 0.185 | 10% | 0.045 |
| Acne | 2% | 0% | 1.000 | 0% | 1.000 | 0% | 1.000 |
| Viral warts | 1% | 0% | 1.000 | 0% | 1.000 | 0% | 1.000 |
| Cold sores | 9% | 4% | 0.699 | 9% | 1.000 | 6% | 1.000 |
| Hearing loss | 18% | 12% | 0.582 | 15% | 0.807 | 16% | 1.000 |
| Tinnitus | 7% | 16% | 0.120 | 12% | 0.300 | 19% | 0.053 |
| Glaucoma | 2% | 0% | 1.000 | 0% | 1.000 | 0% | 1.000 |
| Cataract | 18% | 4% | 0.139 | 3% | **0.034** | 3% | 0.055 |
| Myopia | 10% | 8% | 1.000 | 9% | 1.000 | 6% | 0.745 |
| Age-related macular degeneration | 2% | 8% | 0.118 | 6% | 0.185 | 10% | **0.045** |
| Incontinence | 7% | 8% | 1.000 | 9% | 0.728 | 6% | 1.000 |
| Polycystic ovary syndrome | 1% | 8% | 0.076 | 6% | 0.124 | 6% | 0.107 |
| > 2 comorbidities | 72% | 64% | 0.544 | 65% | 0.503 | 68% | 0.774 |
| > 3 comorbidities | 58% | 52% | 0.729 | 53% | 0.725 | 58% | 1.000 |
| > 4 comorbidities | 44% | 48% | 0.895 | 47% | 0.916 | 52% | 0.578 |
| > 5 comorbidities | 32% | 32% | 1.000 | 32% | 1.000 | 35% | 0.846 |
| > 6 comorbidities | 16% | 28% | 0.260 | 26% | 0.253 | 26% | 0.319 |
| > 7 comorbidities | 12% | 28% | 0.063 | 24% | 0.128 | 26% | 0.076 |
| > 8 comorbidities | 7% | 24% | **0.022** | 18% | 0.113 | 19% | 0.073 |
| > 9 comorbidities | 6% | 12% | 0.209 | 9% | 0.446 | 10% | 0.418 |
| > 10 comorbidities | 2% | 12% | **0.043** | 9% | 0.086 | 10% | 0.070 |

#: Values are *p* values obtained with *Chi*-squared test or Fisher’s exact test comparing each pain group with controls. Values smaller than 0.05 are marked in bold.
