# Supplementary material for: Postoperative joint pain is associated with long-term all-cause mortality after total joint arthroplasty
Source: PLoS One. 2025 Jul 3;20(7):e0327757. doi: 10.1371/journal.pone.0327757 (PMC12225861; doi:10.1371/journal.pone.0327757)
Supplement: Attachment — Submitted filename: PONE-D-25-06222_comments to authors.docx [file pone.0327757.s003.docx]

The manuscript “Postoperative joint pain is associated with long-term all-cause mortality after total joint Arthroplasty” examined the effect of post-operative pain on mortality among patients undergoing TJR. Specific comments and clarifications are below:

1. Abstract (lines 14-16): “… their postoperative joint pain at least one-year after TJA…” Was WOMAC collected at several time points after surgery? Please clarify.
2. Methods, Page 5: Please include the exact duration of the follow-up period (e.g., last date, average # of years of follow-up) and the number of post-operative measurements.
3. Page 5, lines 79-80: Please clarify what post-operative time point was used for the analysis.
4. Page 6: I think that the label ‘sustained pain’ is misleading as it is based on a one time pain reporting based on a question asking for pain in the past 48 hours.

Furthermore, I think that post-operative pain status is not the best variable to accomplish the study objectives, as it does not account for the level of pre-operative pain. I think a variable looking at changes in pain pre- and post- operatively is more appropriate for the study question. Please comment on the choice of the pain variables used.

5. Page 7: Given that comorbidity data was available, I wonder why the models did not control fro comorbidities. Please, clarify
6. Results, Page 7: Given the significant attrition (33%), please expand on the sources of loss to follow-up. For example, how many did not have mortality data and why (e.g., unable to link?), how many did not complete WOMAC? Additionally, how the sample of patients include and excluded differ in terms of basic demographic variables collected. Please include this information in the discussion of the study limitations.
7. Table 1 (knee): the data for age and sex appears to be scrambled.
8. Table 4: Are these estimates from Kaplan-Meier or Cox models?
9. Supplementary Table 1: I suggest including the prevalence of comorbidities. I don’t think that p-values without the prevalence are useful.
