# Supplementary material for: Postoperative joint pain is associated with long-term all-cause mortality after total joint arthroplasty
Source: PLoS One. 2025 Jul 3;20(7):e0327757. doi: 10.1371/journal.pone.0327757 (PMC12225861; doi:10.1371/journal.pone.0327757)
Supplement: Attachment — Submitted filename: Response letter v.2.docx [file pone.0327757.s004.docx]

**Journal requirements:**

When submitting your revision, we need you to address these additional requirements.

1. Please ensure that your manuscript meets PLOS ONE's style requirements, including those for file naming. The PLOS ONE style templates can be found at

<https://journals.plos.org/plosone/s/file?id=wjVg/PLOSOne_formatting_sample_main_body.pdf> and

[https://journals.plos.org/plosone/s/file?id=ba62/PLOSOne_formatting_sample_title_authors_affiliations.pdf](https://track.editorialmanager.com/CL0/https:%2F%2Fjournals.plos.org%2Fplosone%2Fs%2Ffile%3Fid=ba62%2FPLOSOne_formatting_sample_title_authors_affiliations.pdf/1/010f01966974a36c-912e14b1-095e-4c19-800e-8b20c3202ad2-000000/PmzgRTxa2J5SPjhaVT-cnxfIYP3lEg0A35EzO--I87E=208).

Response:

We have reformatted the manuscript to meet the PLOS ONE's style requirements as instructed.

2. PLOS requires an ORCID iD for the corresponding author in Editorial Manager on papers submitted after December 6th, 2016. Please ensure that you have an ORCID iD and that it is validated in Editorial Manager. To do this, go to ‘Update my Information’ (in the upper left-hand corner of the main menu), and click on the Fetch/Validate link next to the ORCID field. This will take you to the ORCID site and allow you to create a new iD or authenticate a pre-existing iD in Editorial Manager.

Response:

ORCID iD for the corresponding author has been validated.

3. Please provide additional details regarding participant consent. In the ethics statement in the Methods and online submission information, please ensure that you have specified (1) whether consent was informed and (2) what type you obtained (for instance, written or verbal, and if verbal, how it was documented and witnessed). If your study included minors, state whether you obtained consent from parents or guardians. If the need for consent was waived by the ethics committee, please include this information.

If you are reporting a retrospective study of medical records or archived samples, please ensure that you have discussed whether all data were fully anonymized before you accessed them and/or whether the IRB or ethics committee waived the requirement for informed consent. If patients provided informed written consent to have data from their medical records used in research, please include this information.

Response:

We have clarified in the Methods section that “informed written consent was obtained from all participants.” (Revised Manuscript with Track Changes, Page 5, lines 81-82)

4. We note that you have indicated that there are restrictions to data sharing for this study. For studies involving human research participant data or other sensitive data, we encourage authors to share de-identified or anonymized data. However, when data cannot be publicly shared for ethical reasons, we allow authors to make their data sets available upon request. For information on unacceptable data access restrictions, please see <http://journals.plos.org/plosone/s/data-availability#loc-unacceptable-data-access-restrictions>.

Before we proceed with your manuscript, please address the following prompts:

a) If there are ethical or legal restrictions on sharing a de-identified data set, please explain them in detail (e.g., data contain potentially identifying or sensitive patient information, data are owned by a third-party organization, etc.) and who has imposed them (e.g., a Research Ethics Committee or Institutional Review Board, etc.). Please also provide contact information for a data access committee, ethics committee, or other institutional body to which data requests may be sent.

b) If there are no restrictions, please upload the minimal anonymized data set necessary to replicate your study findings to a stable, public repository and provide us with the relevant URLs, DOIs, or accession numbers. Please see <http://www.bmj.com/content/340/bmj.c181.long> for guidelines on how to de-identify and prepare clinical data for publication. For a list of recommended repositories, please see <https://journals.plos.org/plosone/s/recommended-repositories>. You also have the option of uploading the data as Supporting Information files, but we would recommend depositing data directly to a data repository if possible.

Please update your Data Availability statement in the submission form accordingly.

Response:

The ethical application for this study that was approved by the Health Research Ethics Authority (HREA) of Newfoundland and Labrador specified that all electronic data for this study shall be stored on a secure Health Science Information and Media Service (HSIMS) server with restricted password-protected access as well as password-protected databases, and the data guardian is Dr. Guangju Zhai. The contact information for HREA of Newfoundland and Labrador is as follows: Email: [info@hrea.ca](mailto:info@hrea.ca), Phone: (709) 864-8871.

Data availability statement:

Data cannot be shared publicly because of the study's ethics application approved by the Health Research Ethics Authority (HREA) of Newfoundland and Labrador (Email: info@hrea.ca). Data are available from the HREA of Newfoundland and Labrador (contact via info@hrea.ca) or corresponding author Dr. Guangju Zhai (contact via gzhai@mun.ca) for researchers who meet the criteria for access to confidential data.

5. Your ethics statement should only appear in the Methods section of your manuscript. If your ethics statement is written in any section besides the Methods, please delete it from any other section.

Response:

The ethics statement now only appears in the Methods section, and the statement written in other sections has been deleted (Revised Manuscript with Track Changes, Page 25, Lines 330-335).

6. Please include captions for your Supporting Information files at the end of your manuscript, and update any in-text citations to match accordingly. Please see our Supporting Information guidelines for more information: <http://journals.plos.org/plosone/s/supporting-information>.

Response:

We have added captions for our Supporting Information files at the end of our manuscript (Revised Manuscript with Track Changes, Page 33, Lines 483-492), and updated in-text citations to match accordingly (Revised Manuscript with Track Changes, Page 8, line 132-133; Page 11, line 166, Page 23, line 289).

Additional Editor Comments:

The reviewers have made comments to improve the manuscript, in particular statistical analysis, interpretation of the results, and implication of study findings.

Response:

We thank the reviewers for their constructive comments and have revised the manuscript accordingly.

Query on May 22, 2025:
1. We dispatched the following message on May 20th, 2025. We note that you have resubmitted your manuscript/updated your statement, but have not replied directly to this query. Additionally, your statement and answer remain the same. Please respond to the following query at your earliest convenience.

Your Data Availability statement currently reads:

"Data cannot be shared publicly because of confidentiality reasons."

Additionally, you indicated that your data is not publicly available, and checked the “No - some restrictions will apply" box when submitting your manuscript.

You also provided some Data Availability information via your Response to Reviewers:

"We have stated in “Availability of data and materials” section that “The datasets used and/or analyzed during the current study are available from the corresponding author on upon reasonable request.” (Revised Manuscript with Track Changes, Page 25, Lines 338-339). The ethical application for this study that was approved by the Health Research Ethics Authority (HREA) of Newfoundland and Labrador specified that all electronic data for this study shall be stored on a secure Health Science Information and Media Service (HSIMS) server with restricted password-protected access as well as password-protected databases, and the data guardian is Dr. Guangju Zhai. The contact information for HREA of Newfoundland and Labrador is as follows: Email: [info@hrea.ca](mailto:info@hrea.ca) <<mailto:info@hrea.ca>>, Phone: (709) 864-8871."

Before we proceed, we’ll require some additional information to ensure your submission adheres to the PLOS ONE Data Availability policy (<https://journals.plos.org/plosone/s/data-availability> <<https://journals.plos.org/plosone/s/data-availability>>)

PLOS asks that authors provide a statement which explains where and how a manuscript's minimal data set can be found. The minimal data set is defined as the data set used to reach the conclusions drawn in the manuscript with related metadata and methods, and any additional data required to replicate the reported study findings in their entirety. This may include: a.) The values behind the means, standard deviations and other measures reported; b.) The values used to build graphs; c.) The points extracted from images for analysis (<https://journals.plos.org/plosone/s/data-availability#loc-minimal-data-set-definition> <<https://journals.plos.org/plosone/s/data-availability#loc-minimal-data-set-definition>>).

If a manuscript's minimal data set cannot be made publicly available, PLOS ONE requires that authors provide a Data Availability statement with specific information to facilitate data access requests. For data that is subject to restrictions, we ask that authors include the following information in their statement:

-The reason for the restriction (i.e., confidentiality of patients, etc.) -The name of the restricting institution -A non-author, institutional point of contact (preferably email) for a data access committee, ethics committee, or other institutional body that other researchers would require to request access to your data. Note that it is not acceptable for an author to be the sole named individual responsible for ensuring data access.

We note that you have provided what appears to be a Data Availability statement associated with restricted data via your Response to Reviewers, but have not updated your statement in the submission system. A manuscript's Data Availability statement should only appear in the submission system, and is generally not included in the body of a manuscript. In your response to this message, please reply to the following queries:

-Can researchers contact the HREA of Newfoundland and Labrador to request access to data via the email address you provided ([info@hrea.ca](mailto:info@hrea.ca) <<mailto:info@hrea.ca>>)?

-Please provide a revised Data Availability statement which includes the information conveyed via your Response to Reviewers. Please ensure that it includes all three pieces of information requested above.

Response:

We have now removed the Data Availability Statement from our manuscript (changed tracked and clean versions).

In the submission system, we have provided a revised Data Availability Statement which includes the information conveyed via our Response to Reviewers, and it includes information on 1) The reason for the restriction (ethics application approved); 2) The name of the restricting institution (the Health Research Ethics Authority (HREA) of Newfoundland and Labrador); 3) A non-author, institutional point of contact (and email) for an ethics committee (the HREA of Newfoundland and Labrador, [info@hrea.ca](mailto:info@hrea.ca)) that other researchers would require to request access to our data (in addition to the corresponding author). Researchers can contact the HREA of Newfoundland and Labrador to request access to data via the email address we provided ([info@hrea.ca](mailto:info@hrea.ca)).

Please see our response to requirement #4 above.

**Reviewers' comments:**

Reviewer's Responses to Questions

**Comments to the Author**

1. Is the manuscript technically sound, and do the data support the conclusions?

The manuscript must describe a technically sound piece of scientific research with data that supports the conclusions. Experiments must have been conducted rigorously, with appropriate controls, replication, and sample sizes. The conclusions must be drawn appropriately based on the data presented.

Reviewer #1: Partly

Reviewer #2: Partly

Reviewer #3: No

2. Has the statistical analysis been performed appropriately and rigorously?

Reviewer #1: Yes

Reviewer #2: No

Reviewer #3: I Don't Know

3. Have the authors made all data underlying the findings in their manuscript fully available?

The [PLOS Data policy](http://track.editorialmanager.com/CL0/http:%2F%2Fwww.plosone.org%2Fstatic%2Fpolicies.action%23sharing/1/010f01966974a36c-912e14b1-095e-4c19-800e-8b20c3202ad2-000000/U9k7t4QSABW5M1FLIUoifJJskHlYdIhd8zseTvGWT6o=208) requires authors to make all data underlying the findings described in their manuscript fully available without restriction, with rare exception (please refer to the Data Availability Statement in the manuscript PDF file). The data should be provided as part of the manuscript or its supporting information, or deposited to a public repository. For example, in addition to summary statistics, the data points behind means, medians and variance measures should be available. If there are restrictions on publicly sharing data—e.g. participant privacy or use of data from a third party—those must be specified.

Reviewer #1: No

Reviewer #2: No

Reviewer #3: Yes

4. Is the manuscript presented in an intelligible fashion and written in standard English?

PLOS ONE does not copyedit accepted manuscripts, so the language in submitted articles must be clear, correct, and unambiguous. Any typographical or grammatical errors should be corrected at revision, so please note any specific errors here.

Reviewer #1: Yes

Reviewer #2: Yes

Reviewer #3: Yes

5. Review Comments to the Author

Please use the space provided to explain your answers to the questions above. You may also include additional comments for the author, including concerns about dual publication, research ethics, or publication ethics. (Please upload your review as an attachment if it exceeds 20,000 characters)

**Reviewer #1:**

The manuscript “Postoperative joint pain is associated with long-term all-cause mortality after total joint Arthroplasty” examined the effect of post-operative pain on mortality among patients undergoing TJR. Specific comments and clarifications are below:

1. Abstract (lines 14-16): “… their postoperative joint pain at least one-year after TJA…” Was WOMAC collected at several time points after surgery? Please clarify.

Response:

The postoperative joint pain was accessed only once for each patient. We have now clarified this in the Abstract (Revised Manuscript with Track Changes, Page 2, Line 19) and Methods (Revised Manuscript with Track Changes, Page 6, Line 85) sections.

2. Methods, Page 5: Please include the exact duration of the follow-up period (e.g., last date, average # of years of follow-up) and the number of post-operative measurements.

Response:

The average follow-up time for postoperative joint pain assessment and the average time from TJA to mortality data extraction were already specified in the Results section (4.0 and 11.0 years after TJA for postoperative joint pain assessment and mortality data extraction, respectively, Manuscript with Track Changes, page 11, line 151, line 154), and we have now added the last follow-up date for postoperative joint pain assessment (July 29, 2019. Manuscript with Track Changes, page 11, lines 151-152) as well as the date of mortality data extraction (June 3, 2024 and January 15, 2025. Manuscript with Track Changes, page 11, lines 154-156). We have also specified in the Methods section that data on postoperative joint pain evaluated by the Western Ontario and McMaster Universities Osteoarthritis Index (WOMAC) Likert 3.0 pain subscale (five questions, Manuscript with Track Changes, page 6, lines 85-89) and three mortality variables were collected (Manuscript with Track Changes, page 6, lines 104-105) after the TJA.

3. Page 5, lines 79-80: Please clarify what post-operative time point was used for the analysis.

Response:

Please see response to comment #2.

4. Page 6: I think that the label ‘sustained pain’ is misleading as it is based on a one-time pain reporting based on a question asking for pain in the past 48 hours.
Furthermore, I think that post-operative pain status is not the best variable to accomplish the study objectives, as it does not account for the level of pre-operative pain. I think a variable looking at changes in pain pre- and post- operatively is more appropriate for the study question. Please comment on the choice of the pain variables used.

Response:

We thank the reviewer for pointing this out. The term “sustained pain” was to describe the status of having joint pain that sustained even after TJA, based on patients’ responses to all five questions in the WOMAC pain subscale (Manuscript with Track Changes, page 6, lines 92-93). All patients in our study cohort had joint diseases with chronic joint pain, hence the pain intensity reported was based on their daily experiences, not just reflecting transient pain. However, the reviewer raised an important point of evaluating postoperative joint pain at multiple timepoints, which could be included in our future study design.

We think both surgery outcome (eg.: postoperative joint pain) and response (eg.: change in joint pain from before to after TJA) could have great impact on patients’ quality of life. In this manuscript, the main aim was to investigate whether postoperative joint pain as the outcome of TJA could be associated with long-term mortality. However, we agree with the reviewer that the association between changes in pain intensity and mortality also needs to be evaluated to broaden our understanding of the impact of TJA on mortality, and this could be a future direction of our study. In addition, the same sustained pain definition has been used in the literature (Costello et al., Individual participant data meta-analysis of metabolomics on sustained knee pain in primary osteoarthritis patients. Rheumatology (Oxford). 2023;62(5):1964-71; Huang et al., Gut Microbiomics of Sustained Knee Pain in Patients With Knee Osteoarthritis. J Rheumatol. 2024 Dec 1;51(12):1218-1225. doi: 10.3899/jrheum.2024-0361).

5. Page 7: Given that comorbidity data was available, I wonder why the models did not control for comorbidities. Please, clarify

Response:

Among the comorbidities we had data on, two were previously reported to be associated with mortality in joint arthroplasty patients: cardiovascular diseases and cancer (Olsen F et al.. The role of bone cement for the development of intraoperative hypotension and hypoxia and its impact on mortality in hemiarthroplasty for femoral neck fractures. Acta Orthop. 2020 Jun;91(3):293-298. doi: 10.1080/17453674.2020.1745510). We have now adjusted for these comorbidities together with age at surgery, sex, and BMI in the Cox proportional hazard model. Results showed that pain while active was still significantly associated with mortality with higher hazard ratios compared to before adjustment when knee and hip patients were analyzed together and separately (hazard ratio (95% confidence interval) = 2.96 (1.90, 4.60), 2.57 (1.52, 4.34), and 4.31 (1.87, 9.90) for knee and hip patients combined, knee patients, and hip patients, respectively). We have revised the Abstract (Manuscript with Track Changes, page 2, lines 25-26, 33-34), Methods (Manuscript with Track Changes, page 7, line 114), Results (Manuscript with Track Changes, page 15, lines 194-196), and Fig 2 to reflect this change.

6. Results, Page 7: Given the significant attrition (33%), please expand on the sources of loss to follow-up. For example, how many did not have mortality data and why (e.g., unable to link?), how many did not complete WOMAC? Additionally, how the sample of patients include and excluded differ in terms of basic demographic variables collected. Please include this information in the discussion of the study limitations.

Response:

We thank the reviewer for the suggestion. We realized that it was not appropriate or accurate to classify all excluded patients as lost to follow-up. We have now provided detailed reasons of exclusion in revised Fig 1 as well as the Results (Manuscript with Track Changes, pages 7-8, lines 124-130). Among the 1086 patients who consented, 95 had bipolar hemiarthroplasty, and TJA for three patients were cancelled, hence these patients were excluded from the analysis. Mortality data for 26 patients could not be linked because they lived outside of NL/Canada. Among the remaining 962 patients, 38 deceased prior to postoperative joint pain assessment, 30 were unable to participate due to health reasons, 27 withdrew consent or unwilling to answer questions, and 140 were unreachable. Among the patients who underwent TJA, the basic demographic variables for the included and excluded patients were compared and results were presented in the Results (Manuscript with Track Changes, page 8, lines 132-133) and S1 Table. There was no difference in sex distribution (p=0.14), but the excluded patients were older and had lower BMI (p<0.001). We have included this information in the discussion of the study limitations (Manuscript with Track Changes, page 24, lines 311-312).

7. Table 1 (knee): the data for age and sex appears to be scrambled.

Response:

This could be due to the file generation during submission. In the version we submitted, the data was not scrambled. We have now listed Table 1 here for your review.

**Table 1. Characteristics of the study participants ^#^**

| **Knee + Hip** | **All (n=731)** | **Control**  **(n=546, 75%)** | **Sustained pain**  **(n=70, 10%)** | ***P-***  **value** | **Pain while active**  **(n=124, 17%)** | ***P-***  **value** | **Pain at rest**  **(n=89, 12%)** | ***P-***  **value** |
| --- | --- | --- | --- | --- | --- | --- | --- | --- |
| **Female (%)** | 55 | 54 | 57 | 0.62 | 56 | 0.62 | 55 | 0.86 |
| **Age at surgery (yrs)** | 64.81 ± 8.77 | 65.69 ± 8.62 | 59.36 ± 9.02 | <0.001** | 61.76 ± 8.93 | <0.001** | 60.00 ± 8.87 | <0.001** |
| **Age at pain assessment (yrs)** | 68.80 ± 8.76 | 69.73 ± 8.59 | 63.16 ± 9.13 | <0.001** | 65.48 ± 8.98 | <0.001** | 63.79 ± 8.90 | <0.001** |
| **BMI (kg/m^2^)** | 33.57 ± 6.95 | 33.39 ± 7.06 | 33.10 ± 6.18 | 0.83 | 33.98 ± 6.59 | 0.21 | 33.13 ± 6.14 | 0.77 |
| **Knee** | **All (n=493)** | **Control**  **(n=361, 73%)** | **Sustained pain**  **(n=44, 9%)** | ***P-***  **value** | **Pain while active**  **(n=89, 18%)** | ***P-***  **value** | **Pain at rest**  **(n=57, 12%)** | ***P-***  **value** |
| **Female (%)** | 64.97 ± 7.95 | 56 | 61 | 0.52 | 60 | 0.57 | 56 | 0.99 |
| **Age at surgery (yrs)** | 57 | 65.64 ± 8.02 | 60.05 ± 7.47 | <0.001** | 62.57 ± 8.02 | 0.003* | 60.83 ± 7.49 | <0.001** |
| **Age at pain assessment (yrs)** | 68.97 ± 7.96 | 69.73 ± 8.02 | 63.80 ± 7.31 | <0.001** | 66.21 ± 7.98 | <0.001** | 64.59 ± 7.39 | <0.001** |
| **BMI (kg/m^2^)** | 33.64 ± 6.91 | 34.43 ± 7.04 | 34.71 ± 6.53 | 0.53 | 35.34 ± 6.63 | 0.14 | 34.67 ± 6.24 | 0.48 |
| **Hip** | **All (n=238)** | **Control**  **(n=185, 78%)** | **Sustained pain**  **(n=26, 11%)** | ***P-***  **value** | **Pain while active**  **(n=35, 15%)** | ***P-***  **value** | **Pain at rest**  **(n=32, 13%)** | ***P-***  **value** |
| **Female (%)** | 50 | 50 | 50 | 0.98 | 49 | 0.90 | 53 | 0.72 |
| **Age at surgery (yrs)** | 64.49 ± 10.27 | 65.79 ± 9.70 | 58.21 ± 11.24 | <0.001** | 59.68 ± 10.76 | <0.001** | 58.52 ± 10.88 | <0.001** |
| **Age at pain assessment (yrs)** | 64.45 ± 10.23 | 69.73 ± 9.64 | 62.07 ± 11.66 | <0.001** | 63.64 ± 11.05 | 0.001* | 62.35 ± 11.08 | <0.001** |
| **BMI (kg/m^2^)** | 31.34 ± 6.50 | 31.33 ± 6.63 | 30.38 ± 4.45 | 0.97 | 30.52 ± 5.09 | 0.77 | 30.38 ± 4.94 | 0.89 |

^#^: Values are the mean ± standard deviation unless indicated otherwise. *P*-values were obtained by the Mann-Whitney *U* test, Student's t-test, or *Chi*-squared test wherever appropriate comparing each pain group with controls; *: *P*-value < 0.05; **: *P*-value < 0.001. BMI: body mass index.

8. Table 4: Are these estimates from Kaplan-Meier or Cox models?

Response:

As stated in the title and legend of Table 4, the estimates were from Kaplan-Meier models: “Table 4. Kaplan-Meier survival estimates”, “P-values, hazard ratios, and confidence intervals were obtained by the Kaplan-Meier survival analysis”. The estimates from Cox models are presented in “Fig 2. Cox survival plot for pain while active in knee and hip patients combined (A), knee patients (B) and hip patients (C)”.

9. Supplementary Table 1: I suggest including the prevalence of comorbidities. I don’t think that p-values without the prevalence are useful.

Response:

We thank the reviewer for the suggestion. This table has now been named S2 Table and we have included the prevalence of comorbidities in this table.

**Reviewer #2:**

Authors present retrospective, secondary analysis of data derived from the Newfoundland Osteoarthritis Study regarding correlation between WOMAC pain scores (classified as ‘sustained pain,’ ‘pain while active,’ and ‘pain at rest) and long-term (11-year) mortality with the idea that post-arthroplasty pain is understudied outside of directly assessing surgical outcomes and that this is an important area of study as chronic pain may have important consequences on health outcomes and mortality. Authors set the stage by reviewing the rising use of arthroplasty surgery as well as previously published work discussing mortality and post-operative pain following hip and knee arthroplasty. After analysis (Kaplan-Meier survival analysis, multivariable Cox proportional hazards regression) the authors conclude that “patient-reported postoperative joint pain, particularly pain while active, could be a valuable predictor of long-term all-cause mortality in TJA patients” and further recommend that this pain be addressed in patients who experience it.

The authors (2 from the division of Genetics & Genomics, 1 from the department of internal medicine, 1 from the department of surgery) having educational training in epidemiology and clinical experience caring for this patient population appear to have the appropriate training to undertake this study.

While successful arthroplasty surgery and the prevention of post-operative pain/mortality are important topics, there are several considerations to be addressed in this manuscript before it will add clearly and positively to the literature.

1. Statistical analysis. Do the authors control for any of the comorbidities listed in the primary analysis? It does not appear that this was the case. It would be important to control for comorbidities (coronary artery disease, chronic obstructive pulmonary disease, peripheral arterial disease, diabetes) which are likely stronger predictors of mortality post-operative mortality in the post-operative setting than pain. It would also be important to control for these comorbidities as some of them may be associated with pain with activity (i.e., CAD, PAD). The authors mention 37 comorbidities being examined with Chi-squared or Fisher’s exact test – however many of these comorbidities are not relevant predictors of mortality. Olsen et al. offer a more concise and relevant list of comorbidities in the citation below.

Olsen F, Hård Af Segerstad M, Nellgård B, Houltz E, Ricksten SE. The role of bone cement for the development of intraoperative hypotension and hypoxia and its impact on mortality in hemiarthroplasty for femoral neck fractures. Acta Orthop. 2020 Jun;91(3):293-298. doi: 10.1080/17453674.2020.1745510. Epub 2020 Apr 2. PMID: 32237931; PMCID: PMC8023921.

Response:

We thank the reviewer for the great reference literature. We had data on two of the comorbidities that were reported to be associated with mortality in hemiarthroplasty for femoral neck fractures in this article, cardiovascular diseases (CVD) and cancer, and we have now adjusted for these two comorbidities together with age at surgery, sex, and BMI in the Cox proportional hazard model. Results showed that pain while active was still significantly associated with mortality with higher hazard ratios compared to before adjustment when knee and hip patients were analyzed together and separately (hazard ratio (95% confidence interval) = 2.96 (1.90, 4.60), 2.57 (1.52, 4.34), and 4.31 (1.87, 9.90) for knee and hip patients combined, knee patients, and hip patients, respectively). We have revised the Abstract (Manuscript with Track Changes, page 2, lines 25-26, 33-34), Methods (Manuscript with Track Changes, page 7, line 114), Results (Manuscript with Track Changes, page 15, lines 194-196), and Fig 2 to reflect this change.

We kept the S2 Table with the 37 comorbidities to compare the disease prevalences between pain and control groups.

2. Selection and interpretation of PROM (WOMAC). The authors bin patients into four groups (‘sustained pain’ versus ‘pain while active’ versus ‘pain at rest’ and controls). Is there any basis for using this approach previously in the literature? The authors citation for utilizing this approach references one of their previous studies which correlated metabolic biomarkers with ‘sustained’ pain. This previous work does not adequately prove that parsing apart the WOMAC measure in this way is valid. How specifically were patients binned? If a patient answered a 1 on both activity (walking, going up/down stairs) questions and a 1 on only a single rest (pain at night, pain while sitting) question were they still binned as a “pain while active” patient? What about the contralateral scenario?

Response:

We did not find any previous literature defining characteristics of pain based on WOMAC pain subscale questions. We defined these three types of pain based on the different aspects of patients’ daily activities that could be affected by pain reflected by the five pain subscale questions to study what type of pain could be associated with mortality after TJA. If a patient answered a 1 on both activity (walking, going up/down stairs) questions, then this patient was classified as “pain while active” patient regardless of what the answers were for other questions. In other words, what all patients had in common in this group was that they all had pain while doing activities. The same classification method was used for “pain at rest” patients. If a patient answered a 1 on pain at night and a 1 on pain while sitting, then this patient was classified as “pain at rest” patient regardless of what the answers were for other questions. These same case definitions were also used in another publication (Huang et al., Gut Microbiomics of Sustained Knee Pain in Patients With Knee Osteoarthritis. J Rheumatol. 2024 Dec 1;51(12):1218-1225. doi: 10.3899/jrheum.2024-0361).

3. Discussion/interpretation of results. The conclusion that ‘Efforts should be taken to address such pain in these patients, especially in those with preexisting cardiac/circulatory conditions and/or neoplasms to potentially reduce increased mortality’ highlights the disconnect between the studies methodology, conclusions, and the literature. This study’s findings are too preliminary to make a recommendation such as this. Further, the recommendation is disconnected form the clinical literature as arthroplasty surgeons are always trying to treat their patient’s pain – so they are already doing this. The introduction presents a more compelling interpretation of the goals for the data: ‘It is important to identify indicators for mortality to better inform patients, enhance patient care algorithms, and monitor surgical care systems.’ The discussion could also be made more concise.

Response:

We thank the reviewer for pointing this out. We have revised the Discussion and Conclusions sections to make them more concise (Manuscript with Track Changes, pages 23-24, lines 300-308, 325-328). We agree that arthroplasty surgeons are always trying to treat their patient’s pain, while our findings raised the question whether the same treatment strategy should be utilized for patients with different types of postoperative pain (e.g.: pain at rest vs. pain while active). Given the previous evidence on the associations between pain, physical activity, depression, and cardiovascular diseases and cancers, our findings might open new topics for discussion: whether treating other conditions closely related to pain and helping these patients resume regular daily activities should also be important parts in the therapeutic regime for TJA patients, a question not only involves the health care system but also social support. The findings could help inform patients in postoperative care and develop more targeted patient care algorithms in TJA patients.

Other Specifics:
-70: Is there a citation that can be included that can link to the study protocol or provide more detail on the index study. It is outside the scope of this specific article but readers should be able to easily access what the goal of the original study is, what the inclusion/exclusion criteria were, etc.

Response:

There are some citations using the Newfoundland Osteoarthritis Study (NFOAS) cohort, but none provides more detailed study protocol. As stated in the Methods, recruited patients aged 20 years or older who had joint arthroplasty between 30/11/2011 and 05/02/2017 in St. Clare’s Mercy Hospital and Health Science Centre General Hospital in St. John’s, Newfoundland and Labrador. Patients were recruited consecutively. We have now clarified this in the Methods section (Manuscript with Track Changes, page 5, lines 79-80).

-79: Is there a citation that can be included which provides data on the validity of the WOMAC?

Response:

A citation (#27) on the validity of the WOMAC was already included in the Discussion section.

McConnell S, Kolopack P, Davis AM. The Western Ontario and McMaster Universities Osteoarthritis Index (WOMAC): a review of its utility and measurement properties. Arthritis Rheum. 2001;45(5):453-61.

-117-118: Not ideal loss to follow-up.

Response:

Please see response to Reviewer #1, comment #6.

-97-99 + page 16 ‘postoperative joint pain and COD’ section: cause of death data was only available for 10% of the original sample.

Response:

As stated in the Results section, among the 727 patients (731 records) included in the analysis, 129 deceased at the time of mortality data extraction, and COD data was available for 78 (60%, Manuscript with Track Changes, page 11, line 166). COD data for patients who deceased after Dec 31, 2022 was unavailable due to the timeline of data release to the Newfoundland and Labrador Centre for Health Information Mortality System. We have acknowledged this as a limitation of the study (Manuscript with Track Changes, page 11, lines 156-158).

**Reviewer #3:**

I thank the authors and the editorial team for the opportunity to review their work on the association of joint pain one year postoperatively and mortality. I think I do understand how this study came to be conceptually, but have difficulties to understand the conclusions drawn from the results. Foremost: What would your biological explanation be for the established association? Would mortality go down if we would give patients more pain killers? Why did your model not include comorbidity even though you had access to this important information? In summary, I am afraid that I do not consider that this study adds to the literature in a meaningful way.

Response:

We thank the reviewer for the comments. As this study was an epidemiological study, we did not investigate the underlying biological mechanisms. What was found in this study was the positive association between postoperative joint pain, specifically pain while active, and long-term mortality, not a causal relationship between these two. Therefore, we did not suggest that giving patients more pain killers would lower mortality rate. We have revised the Discussion and Conclusions sections to make it clearer (Manuscript with Track Changes, pages 23-24, lines 300-308, 325-328). We have now included comorbidities that have been previously reported to be associated with mortality in joint arthroplasty patients: cardiovascular diseases and cancer (Olsen F et al.. The role of bone cement for the development of intraoperative hypotension and hypoxia and its impact on mortality in hemiarthroplasty for femoral neck fractures. Acta Orthop. 2020 Jun;91(3):293-298. doi: 10.1080/17453674.2020.1745510) in our Cox proportional hazard model. Results showed that pain while active was still significantly associated with mortality with higher hazard ratios compared to before adjustment. Please see response to Reviewer #1, comment #5. We agree that arthroplasty surgeons are always trying to treat their patient’s pain, while this study called for even more attention on patients who have joint pain during activities after TJA, and opened new topics for discussion such as whether treating postoperative joint pain itself was enough, whether treating other conditions closely related to pain, providing social support, and helping these patients resume regular daily activities should also be important parts in the therapeutic regime for TJA patients (Manuscript with Track Changes, pages 23-24). We think this does make this study add to the literature in a meaningful way.

6. PLOS authors have the option to publish the peer review history of their article ([what does this mean?](https://track.editorialmanager.com/CL0/https:%2F%2Fjournals.plos.org%2Fplosone%2Fs%2Feditorial-and-peer-review-process%23loc-peer-review-history/1/010f01966974a36c-912e14b1-095e-4c19-800e-8b20c3202ad2-000000/Q38krYv7APQ_xdWrDCXaEwGAwI0zp4icanod2yrO7fE=208)). If published, this will include your full peer review and any attached files.

Response:

Yes.

If you choose “no”, your identity will remain anonymous but your review may still be made public.


**Do you want your identity to be public for this peer review?** For information about this choice, including consent withdrawal, please see our [Privacy Policy](https://track.editorialmanager.com/CL0/https:%2F%2Fwww.plos.org%2Fprivacy-policy/1/010f01966974a36c-912e14b1-095e-4c19-800e-8b20c3202ad2-000000/yoNkoJQvbLL2uTki2IuZfkHqTwuHcr4FoQi_V1Z8xQw=208).

Reviewer #1: No

Reviewer #2: No

Reviewer #3: **Yes:** Anders Brüggemann, MD, PhD
