# Supplementary material for: Postoperative joint pain is associated with long-term all-cause mortality after total joint arthroplasty
Source: PLoS One. 2025 Jul 3;20(7):e0327757. doi: 10.1371/journal.pone.0327757 (PMC12225861; doi:10.1371/journal.pone.0327757)
Supplement: Attachment — Submitted filename: Response letter.v.3.docx [file pone.0327757.s005.docx]

**Journal Requirements:**

Please review your reference list to ensure that it is complete and correct. If you have cited papers that have been retracted, please include the rationale for doing so in the manuscript text, or remove these references and replace them with relevant current references. Any changes to the reference list should be mentioned in the rebuttal letter that accompanies your revised manuscript. If you need to cite a retracted article, indicate the article’s retracted status in the References list and also include a citation and full reference for the retraction notice.

**Response:**

We have reviewed the reference list and corrected reference #27. In PubMed, the journal name for this article is listed as “Arthritis Rheum”, which was presented as the journal name for this reference when we added it using PMID number. However, the full text shows that this article was published in “Arthritis Care & Research”. The volume, issue, and page numbers were correct. We have corrected the journal name of this reference. We did not cite any papers that have been retracted. The reference list is now complete and correct.

**Additional Editor Comments:**

The authors have addressed the reviewers' comments appropriately. One reviewer requested a minor revision by adding a limitation.

**Response:**

We thank the editor and reviewers for approving the changes we have made according to the editor and reviewers’ constructive comments. We have added a limitation as suggested by reviewer #1.


**Reviewers' comments:**

**Reviewer's Responses to Questions**

**Comments to the Author**

1. If the authors have adequately addressed your comments raised in a previous round of review and you feel that this manuscript is now acceptable for publication, you may indicate that here to bypass the “Comments to the Author” section, enter your conflict of interest statement in the “Confidential to Editor” section, and submit your "Accept" recommendation.

Reviewer #1: All comments have been addressed

2. Is the manuscript technically sound, and do the data support the conclusions?

The manuscript must describe a technically sound piece of scientific research with data that supports the conclusions. Experiments must have been conducted rigorously, with appropriate controls, replication, and sample sizes. The conclusions must be drawn appropriately based on the data presented.

Reviewer #1: Yes

3. Has the statistical analysis been performed appropriately and rigorously?

Reviewer #1: Yes

4. Have the authors made all data underlying the findings in their manuscript fully available?

The [PLOS Data policy](http://track.editorialmanager.com/CL0/http:%2F%2Fwww.plosone.org%2Fstatic%2Fpolicies.action%23sharing/1/010f01977d23e846-a8b80ad4-1652-4058-8129-d76f6a1ba78b-000000/THYTaYBTcJdvw5Te8FBtQpMDP4bGmFMOi3qknScMAiY=215) requires authors to make all data underlying the findings described in their manuscript fully available without restriction, with rare exception (please refer to the Data Availability Statement in the manuscript PDF file). The data should be provided as part of the manuscript or its supporting information, or deposited to a public repository. For example, in addition to summary statistics, the data points behind means, medians and variance measures should be available. If there are restrictions on publicly sharing data—e.g. participant privacy or use of data from a third party—those must be specified.

Reviewer #1: Yes

5. Is the manuscript presented in an intelligible fashion and written in standard English?

PLOS ONE does not copyedit accepted manuscripts, so the language in submitted articles must be clear, correct, and unambiguous. Any typographical or grammatical errors should be corrected at revision, so please note any specific errors here.

Reviewer #1: Yes

6. Review Comments to the Author

Please use the space provided to explain your answers to the questions above. You may also include additional comments for the author, including concerns about dual publication, research ethics, or publication ethics. (Please upload your review as an attachment if it exceeds 20,000 characters)

Reviewer #1: I thank the authors for addressing my comments thoroughly. My only remaining comment, though, is that I suggest adding a sentence to the limitations section pointing out that the analysis did not account for pre-operative level of pain.

**Response:**

We thank the reviewer for the suggestion. We have added this limitation in the revised manuscript (Revised Manuscript with Track Changes, Page 24, Lines 311-315).

7. PLOS authors have the option to publish the peer review history of their article ([what does this mean?](https://track.editorialmanager.com/CL0/https:%2F%2Fjournals.plos.org%2Fplosone%2Fs%2Feditorial-and-peer-review-process%23loc-peer-review-history/1/010f01977d23e846-a8b80ad4-1652-4058-8129-d76f6a1ba78b-000000/j_cpopDNKMhhax4HYJBDOXKeM4Ve9gcaeqyHMUWUPZE=215)). If published, this will include your full peer review and any attached files.


If you choose “no”, your identity will remain anonymous but your review may still be made public.


**Do you want your identity to be public for this peer review?** For information about this choice, including consent withdrawal, please see our [Privacy Policy](https://track.editorialmanager.com/CL0/https:%2F%2Fwww.plos.org%2Fprivacy-policy/1/010f01977d23e846-a8b80ad4-1652-4058-8129-d76f6a1ba78b-000000/mgeQ7OUIerizNQQWtR6Ls-ehflJMK1IqBDDtANni3KY=215).

Reviewer #1: No
